# Draft Genome Sequence of *Lactobacillus plantarum* wikim18, Isolated from Korean Kimchi

**DOI:** 10.1128/genomeA.00467-14

**Published:** 2014-05-22

**Authors:** Ja Young Jang, Hyeong In Lim, Hae Woong Park, Hak-Jong Choi, Tae-Woon Kim, Miran Kang, Jong-Hee Lee

**Affiliations:** World Institute of Kimchi, Gwangju, Republic of Korea

## Abstract

This report describes the draft genome sequence of *Lactobacillus plantarum* strain wikim18, isolated from the traditional Korean food kimchi. The reads generated by Ion Torrent PGM were assembled into 327 contigs. RAST annotation of the genome revealed 12 tRNAs and 3,316 protein-coding gene sequences.

## GENOME ANNOUNCEMENT

*Lactobacillus plantarum* strain wikim18 was primarily isolated in 2013 at the World Institute of Kimchi, Republic of Korea, upon investigation of a bacterial community during kimchi fermentation. *L. plantarum* is a Gram-positive bacterium that is classified as a lactic acid bacterium (LAB) group associated with many fermented foods, including the traditional Korean food kimchi (1, 2).

During kimchi fermentation, the LAB response suppresses the growth of aerobe bacteria by acidifying the environment. At the late stages of kimchi fermentation, the pH is lowered to 4.0 and the lactic acid bacterium community is simplified. *L. plantarum* is a major lactic acid bacterium found in the late stages of kimchi fermentation due to the acid tolerance phenotype. Therefore, it is of great interest to undercover the genome sequences of *L. plantarum*.

*L. plantarum* strain wikim18 was selected from homemade kimchi using MRS media and the primary taxon was identified by 16S rRNA gene sequencing. The sequence has 99% similarity with *L. plantarum* WCFS1 (NR_075041.1) and *L. plantarum* strain NRRL B-14768 (NR_042394.1) 16S rRNA genes.

DNA was extracted from pure cultures by taking a single colony into MRS media and incubating it anaerobically at 30°C for 14 h. Genomic DNA was isolated with the QIAamp DNA extraction kit (Qiagen). Quality control of all libraries was performed on the Agilent Bioanalyzer using a high sensitivity chip. For determination of the complete genome sequence of *L. plantarum* strain wikim18, a fragment library was sequenced with Ion Torrent PGM using a 318 chip. After trimming the adaptor sequences, 6,873,372 reads were produced with 95.45% trimming efficiency. The sequence was assembled *de novo* into 327 contigs and mapped to references using the CLC genomics workbench (v7.0.3) (http://www.clcbio.com/files/whitepapers/whitepaper-denovo-assembly-4.pdf). The total size of the assembly is 3,353,389 bp, with an average length of 10,255 bp and a G+C content of 44.3%. Mapping to the reference sequence matches 82.43% with *L. plantarum* WCFS1 (NC_004567.2) and 86.16% with *L. plantarum* JDM1 (NC_012984.1).

The genome sequence was annotated using the RAST (3) genome annotation server. The contigs of *L. plantarum* wikim18 were annotated and found to have 336 subsystems, 3,316 coding sequences, and 66 RNAs.

The RAST server identified the genes present in the test strain genome, but not in the reference genome. Among these genes were those encoding a GlpG protein, glyceraldehyde-3-phosphate ketol-isomerase, and inosose isomerase associated with sugar alcohol production. The draft genome sequence of *L. plantarum* described in this report will help the understanding of future kimchi fermentation.

### Nucleotide sequence accession numbers.

This whole-genome shotgun project has been deposited at DDBJ/EMBL/GenBank under the accession no. JMEL00000000. The version described in this paper is version JMEL01000000.
